# Effects of Psychological Stress on Hypertension in Middle-Aged Chinese: A Cross-Sectional Study

**DOI:** 10.1371/journal.pone.0129163

**Published:** 2015-06-04

**Authors:** Bo Hu, Xiaoyu Liu, Sufeng Yin, Hongmin Fan, Fumin Feng, Juxiang Yuan

**Affiliations:** 1 School of Public Health and the Hebei Province Key Laboratory of Occupational Health and Safety for Coal Industry, Hebei United University, Tangshan, Hebei Province, China; 2 Department of Respiratory Medicine, Kailuan General Hospital, Hebei United University, Tangshan, Hebei Province, China; Shanghai Institute of Hypertension, CHINA

## Abstract

We examined the effect and relative contributions of different types of stress on the risk of hypertension. Using cluster sampling, 5,976 community-dwelling individuals aged 40–60 were selected. Hypertension was defined according to the Seventh Report of the Joint National Committee, and general psychological stress was defined as experiencing stress at work or home. Information on known risk factors of hypertension (e.g., physical activity levels, food intake, smoking behavior) was collected from participants. Logistic regression analysis was used to determine the associations between psychological stress and hypertension, calculating population-attributable risks and 95% confidence intervals (CIs). General stress was significantly related to hypertension (odds ratio [OR] = 1.247, 95% CI [1.076, 1.446]). Additionally, after adjustment for all other risk factors, women showed a greater risk of hypertension if they had either stress at work or at home: OR = 1.285, 95% CI (1.027, 1.609) and OR = 1.231, 95% CI (1.001, 1.514), respectively. However, this increased risk for hypertension by stress was not found in men. General stress contributed approximately 9.1% (95% CI [3.1, 15.0]) to the risk for hypertension. Thus, psychological stress was associated with an increased risk for hypertension, although this increased risk was not consistent across gender.

## Introduction

Non-communicable cardiovascular diseases (CVDs) are predicted to be the leading cause of death and disability globally by 2020 [1]. The number of hypertensive adults will reach 1.5 billion—approximately 30% of the world population—by 2025 [2]. Importantly, hypertension—or high blood pressure (BP)—despite being a highly modifiable antecedent for CVDs, is responsible for more deaths worldwide than any other antecedent, including tobacco use, obesity, and lipid disorders [3]. Hypertension is a major health problem globally due to its magnitude and associated risks, how difficult it is to manage, its high medical and social costs, and the fact that it causes severe cardiovascular and renal complications [4]. There are many risk factors for hypertension, including older age, having a family history of the condition, being overweight or obese, low physical activity levels, and tobacco use. Nevertheless, the etiology of hypertension remains poorly understood, as the genetic and behavioral factors known to be involved leave a substantial portion of variability in outcomes unexplained.

In recent decades, technological revolutions in agriculture and industry have dramatically changed lifestyles and social interactions, and generate a substantial amount of psychosocial stress.

A set of behavioral and hormonal responses were induced in individuals with these stressful experiences for adapting to the physical and social environment. Researchers have hypothesized that psychological stress is an important risk factor for essential hypertension [5, 6], and there is some evidence from prospective studies on an association between psychosocial stress—primarily daily stress, such as that experienced at work—and elevated BP [7, 8]. Moreover, some studies mentioned that associations of psychological stress and elevated BP were different by gender [8].

The effects of chronic stress are being investigated in a number of domains—including work, marriage, and low socioeconomic status. Associations between these domains and BP outcomes have been reported, but the evidence varies, and ongoing exposure to stress may be more plausibly linked to sustained BP elevations and incidence of hypertension [9]. In addition, to our knowledge, few studies have evaluated the relationships of a variety of types of stress with hypertension, meaning that this relationship remains inconclusive. Therefore, it would be important to obtain credible evidence on the specifics of the relationship between psychosocial stress and hypertension.

In the present study, we comprehensively evaluated the associations between stress and hypertension in a large-scale cross-sectional study of a middle-aged population from Mainland China to address the above-mentioned gap in the literature. The interactions between gender and different types of stress were explored and the population-attributable risk (PAR) of stress was calculated using a comprehensive and standardized collection of various risk factors of hypertension.

## Materials and Methods

### Ethics Statement

The ethics committees at Hebei United University in China approved the study, and all participants supplied written informed consent.

### Sampling

From 2009 to 2011, community-based participants in urban and rural areas in the Chinese province of Hebei were recruited via cluster sampling. Four representative urban and rural communities were selected and all individuals 40–60 years of age living in the selected communities more than 5 years were interviewed and participated voluntarily. Of these, 12,201 residents met the inclusion criteria, and 10,236 participants completed the questionnaire and laboratory tests (response rate: 83.9%). In this analysis, participants were excluded if they had a history of hypertension, CVD, or stroke; had taken antihypertension/lipid-lowering agents regularly in the previous month; or had missing stress data (from work or home) or data for one of the main demographic variables (age or gender). Therefore, 5,976 participants (2,359 men and 3,617 women) were included in this analysis.

#### Data collection

Data were collected at examination centers in local health stations or community clinics in participants’ residential areas. The quality of data collection was maintained by using standardized protocols and centralized training of assistants. All data were electronically entered at each center into a customized database programmed with the appropriate ranges and were checked for consistency using quality control measures.

#### Questionnaire survey

Structured and pre-test questionnaires were administered in face-to-face interviews. The first part of the survey covered basic participant characteristics, including age, gender, ethnicity, marital status, education, occupation, tobacco use, physical activity, and diet. The second part asked about respondents’ health information, including chronic diseases, current illness, and history and treatment of hypertension, diabetes mellitus, CVDs, and stroke. At the same time, information about the family history of these chronic diseases was also collected.

Our measure of psychological stress was designed using the Composite International Diagnostic Interview Short Form for Major Depression [10, 11]. Psychological stress was assessed with two items—one relating to stress at work and the other to stress at home. Stress was defined for participants as feeling irritable or filled with anxiety or having sleeping difficulties as a result of the conditions at work or at home within the previous year. For each item, participants had four response options representing categories of stress: no experience of stress, some periods of stress, several periods of stress, and persistent stress.

#### Physical examination and laboratory tests

The same examiner performed standard, simple physical examinations for each participant; the measurements included height, weight, waist and hip circumference, and heart rate. Body weight and height were measured during the examination. The body mass index (BMI) was defined as weight (kg) divided by squared height (m^2^). Overweight was defined as a BMI of 24.0 to 27.9 kg/m^2^ and obesity as a BMI of 28.0 kg/m^2^ or more, as per World Health Organization definitions [12].

During the clinical examination, trained and certified observers collected BP and anthropometric measurements using standard protocols and techniques. BP was assessed using the Omron HEM-757 automatic digital monitor (Omron Healthcare). Three BP readings were obtained with the participant in a seated position after 5 min of rest; the mean rate was used in the statistical analysis. Participants were advised to avoid cigarette smoking, alcohol, caffeinated beverages, and exercise for at least 30 min before their BP was taken.

Blood samples were collected in the early morning before participants had eaten. These samples were immediately stored in ice-filled insulated containers and were centrifuged, aliquoted, and stored in -70°C freezers within 2 hours of collection. Plasma glucose was measured with a modified hexokinase enzymatic method. Concentrations of high-density lipoprotein cholesterol (HDL-C) and triglycerides (TG) were assessed enzymatically with commercially available reagents [13].

### Statistical methods

#### Definition of variables

According to the Seventh Report of the Joint National Committee on the Prevention, Detection, Evaluation, and Treatment of High Blood Pressure [14], hypertension was defined as systolic BP (SBP) of 140 mmHg or greater, or diastolic BP (DBP) of 90 mmHg or greater. General stress was constructed by merging stress at work and at home. Participants were divided into two groups (Yes vs. No)—those with stress experience (either at work or at home) and those with no stress experience at all. We used these categories for all subsequent analyses.

Marital status was classified into six categories: never married, currently married, common law marriage/cohabiting, widowed, separated, and divorced. We then combined these categories into two higher-order categories according to the amount of social support present: thus, widowed, divorced, and separated were combined into “single,” while never married, currently married, and common law marriage/cohabiting were combined into “not single.” Education was divided into five categories: illiterate, primary school, junior high school, high school/secondary specialized school/secondary technical school, and junior college/university. These categories were collapsed into three categories: primary (illiterate and primary school), secondary (junior high school and high school/secondary specialized school/secondary technical school), and senior (junior college/university).

Tobacco use was assessed using 3 categories by participant self-report: never, formerly, and current. According to the influences of genetic factors, family history was defined as at least one parent having a history of hypertension.

The long form of the International Physical Activity Questionnaire [15] was used to assess the physical activity of respondents in the following domains: domestic and gardening, transport-related, leisure-time, and work-related activities. The three levels of physical activity were low, moderate, and vigorous, and were based on the metabolic equivalent task score calculated from the estimated energy cost of each activity.

Consumption of vegetables and fruits was assessed by asking participants the amount of all types of vegetables or fruits that were part of the Chinese diet they had consumed in the past year (calculation method = quantity per time × consumption times). The fatty foods category included all types of meat, eggs, milk, and fish, and the calculation method for the amount consumed was the same as for the vegetables and fruits. Information about diet and food intake was collected from self-reports.

#### Statistical analysis

Baseline characteristics were described as means (SD) for continuous variables and frequencies for categorical variables. Categorical variables were compared with the chi-square test. Continuous variables were compared with t-tests or appropriate nonparametric tests when distributional assumptions were in doubt.

Multivariate logistic regression models were used to measure associations between different types of stress and hypertension after adjusting for other risk factors including age, BMI, family history of hypertension, tobacco use, intake of fatty foods, intake of vegetables and fruits, blood biochemical indicators (fasting plasma glucose, TG, and HDL-C), education, marital status, and physical activity. The strength and direction of the associations were indicated by odds ratios (OR) and corresponding 95% confidence intervals (CI). As is common in multivariate models, dummy variables were created for polytomous variables. Log transformations were conducted according to the distributions of the independent variables in the multivariate analysis. Then, a sensitivity analysis was conducted using two different datasets—the first (Dataset 1) was the whole population from which we drew our sample, with excluding participants who had missing stress data (from work or home) or data for one of the main demographic variables (age or gender); the second (Dataset 2) excluded participants who had missing stress data (from work or home) or data for one of the main demographic variables (age or gender), or who had taken antihypertension/lipid-lowering agents regularly. The same factors as in the main study were adjusted in the logistic regression analyses using the two datasets.

PARs and 95% CIs were calculated using a method based on unconditional logistic regression [16] and adjusted for other risk factors (including age, BMI, family history of hypertension, tobacco use, intake of fatty foods, intake of vegetables and fruits, blood biochemical indicators [fasting plasma glucose, TG, and HDL-C], education, marital status, and physical activity).

In addition, the interactions of gender and different types of stress were investigated using logistic models. The Mantel–Haenszel chi-square test was used as a linear trend test for ordinal variables.

A P-value of 0.05 or less was considered statistically significant, and all statistical tests were two-sided. All statistical analyses were performed using SAS 9.13 software.

## Results

### Participants’ characteristics

The data of 5,976 participants (2,359 or 39.47% male) were included in the analysis. The mean (SD) age was 49.95 (5.59) years and about 15.29% of the sample participants had hypertension. About 38.45% participants reported general stress. SBP, DBP, and rates of hypertension were higher in the general stress group than in the no general stress group. Regarding the other risk factors for hypertension, age and proportions of men, single participants, current smokers, participants with a family history of hypertension, and participants with “low” physical activity were higher in the general stress group; however, TG, intake of fatty foods, and intake of vegetables and fruits were higher in the no general stress group. The demographic variables are summarized in Table 1.

**Table 1:** Demographic characteristics of participants by general stress.

	Total (n = 5976)	General stress		P^a^
		Yes (n = 2298)	No (n = 3678)	
**Blood pressure**				
**SBP (mmHg)**	128.15 ± 18.94	128.49 ± 18.87	127.96 ± 18.99	0.292
**DBP (mmHg)**	81.05 ± 11.04	81.50 ± 11.29	80.69 ± 10.88	0.006
**Hypertension (Yes, %)**	914 (15.29)	376 (16.36)	538 (14.63)	0.070
**Other risk factors for hypertension**				
**Age (years)**	49.95 ± 5.59	49.22 ± 5.63	50.41 ± 5.51	<.0001
**Gender (male, %)**	2359 (39.47)	959 (41.73)	1400 (38.06)	0.005
**BMI (kg/m^2^)**	24.62 ± 3.92	24.54 ± 4.07	24.67 ± 3.82	0.218
**Fatty food intake (g/d)** ^**b**^	135.18 (77.74, 202.36)	113.05 (62.58, 188.22)	148.26 (88.66, 209.59)	<.0001
**Vegetable and fruit intake (g/d)** ^**b**^	500.00 (339.73, 639.86)	482.19(333.56,624.79)	500.00(345.34, 654.25)	0.001
**TG (mmol/L)** ^**b**^	1.19 (0.84, 1.70)	1.16 (0.83, 1.63)	1.20 (0.85, 1.74)	0.043
**HDL-C (mmol/L)**	1.35 ± 0.32	1.35 ± 0.31	1.35 ± 0.33	0.457
**Fasting plasma glucose (mmol/L)**	5.57 ± 0.97	5.54 ± 0.97	5.58 ± 0.97	0.115
**Education**				
**Primary**	1471 (24.71)	554 (24.18)	917 (25.05)	0.255
**Secondary**	4280 (71.91)	1669 (72.85)	2611 (71.32)	
**Senior**	201 (3.38)	68 (2.97)	133 (3.63)	
**Marital status**				
**Single**	229 (3.83)	106 (4.61)	123 (3.34)	0.013
**Tobacco use**				
**Never**	4100 (70.39)	1520 (67.89)	2580 (71.95)	0.003
**Formerly**	148 (2.54)	56 (2.50)	92 (2.57)	
**Current**	1577 (27.07)	663 (29.61)	914 (25.49)	
**Family history of hypertension**				
**Yes**	411 (6.88)	177 (7.70)	234 (6.36)	0.046
**Physical activity**				
**Low**	561 (9.39)	263 (11.44)	298 (8.10)	<.0001
**Moderate**	2641 (44.19)	999 (43.47)	1642 (44.64)	
**Vigorous**	2774 (46.42)	1036 (45.08)	1738 (47.25)	

BMI: body mass index; SBP: systolic blood pressure; DBP: diastolic blood pressure; TG: triglycerides; HDL-C: high-density lipoprotein cholesterol.

^a^P-value of the comparison by general stress.

^b^Median values (Q1, Q3).

### Crude rates of hypertension by different types of stress and gender

The number of participants with hypertension was 914 (15.29%) in the total sample, and 410 (17.38%) and 504 (13.93%) men and women had it, respectively. The crude rates of hypertension in the participants with general stress, stress at work, and stress at home were higher than the rate in the participants without stress (16.36% vs. 14.63%, 16.59% vs. 14.77%, and 16.62% vs. 14.64%, respectively). Similar results were found for both men and women. However, only the rates of stress at home in the total sample were significantly different (P = 0.046) (Table 2).

**Table 2:** The crude rates of hypertension for different types of stress by gender.

	Total (n = 5976)			Men (n = 2359)			Women (n = 3617)		
	n	Hypertension (%)	P	n	Hypertension (%)	P	n	Hypertension (%)	P
**General stress**									
** No**	3678	538 (14.63)	0.070	1400	233 (16.64)	0.254	2278	305 (13.39)	0.217
** Yes**	2298	376 (16.36)		959	177 (18.46)		1339	199 (14.86)	
**Stress at work**									
** No**	4258	629 (14.77)	0.077	1549	263 (16.98)	0.477	2709	366 (13.51)	0.204
** Yes**	1718	285 (16.59)		810	147 (18.15)		908	138 (15.20)	
** Some periods**	1573	256 (16.27)		738	131 (17.75)		835	125 (14.97)	
** Several periods**	45	8 (17.78)		21	4 (19.05)		24	4 (16.67)	
** Persistent**	100	21 (21.00)		51	12 (23.53)		49	9 (18.37)	
**Stress at home**									
** No**	4002	586 (14.64)	0.046	1560	262 (16.79)	0.295	2442	324 (13.27)	0.095
** Yes**	1974	328 (16.62)		799	148 (18.52)		1175	180 (15.32)	
** Some periods**	1812	300 (16.56)		723	134 (18.53)		1089	166 (15.24)	
** Several periods**	48	7 (14.58)		27	4 (14.81)		21	3 (14.29)	
** Persistent**	114	21 (18.42)		49	10 (20.41)		65	11 (16.92)	

P-values represent the comparisons between each pair of “No” and “Yes” groups by gender and type of stress.

### Odds ratios for hypertension by different types of stress

An unconditional logistic regression method was used to test the association between stress and hypertension while adjusting for other factors, which included age, BMI, family history of hypertension, tobacco use, intake of fatty foods, intake of vegetables and fruits, blood biochemical indicators (fasting plasma glucose, TG, and HDL-C), education, marital status, and physical activity (Table 3). General stress was associated with a higher odds of hypertension in the total sample (OR = 1.247, 95% CI [1.076, 1.446]), and for men and women (OR = 1.250, 95% CI [1.003, 1.558]) and OR = 1.229, 95% CI [1.005, 1.503], respectively). In the total sample, stress at work or home was associated a higher odds of hypertension compared to having stress at neither work nor home (OR = 1.194, 95% CI [1.017, 1.401] and OR = 1.202, 95% CI [1.032, 1.401], respectively). Women showed similar results (OR = 1.285, 95% CI [1.027, 1.609] and OR = 1.231, [1.001, 1.514], respectively). However, for men, the higher odds were not significant (OR = 1.087, 95% CI [0.864, 1.367] and OR = 1.154, 95% CI [0.919, 1.450], respectively).

**Table 3:** Adjusted odds ratios (ORs)^a^ for hypertension by the different types of stress.

	Total ^b^ (n = 5976)		Men (n = 2359)		Women (n = 3617)	
	n	OR (95% CI)	n	OR (95% CI)	n	OR (95% CI)
**General stress**						
** No**	3678	1	1400	1	2278	1
** Yes**	2298	1.247 (1.076, 1.446)	959	1.250 (1.003, 1.558)	1339	1.229 (1.005, 1.503)
**Stress at work**						
** No**	4258	1	1549	1	2709	1
** Yes**	1718	1.194 (1.017, 1.401)	810	1.087 (0.864, 1.367)	908	1.285 (1.027, 1.609)
** Some periods**	1573	1.156 (0.978, 1.365)	738	1.062 (0.836, 1.350)	835	1.230 (0.975, 1.551)
** Several periods**	45	1.191 (0.538, 2.636)	21	0.857 (0.273, 2.694)	24	1.588 (0.529, 4.769)
** Persistent**	100	1.557 (0.935, 2.594)	51	1.358 (0.680, 2.711)	49	1.707 (0.799, 3.649)
**Stress at home**						
** No**	4002	1	1560	1	2442	1
** Yes**	1974	1.202 (1.032, 1.401)	799	1.154 (0.919, 1.450)	1175	1.231 (1.001, 1.514)
** Some periods**	1812	1.184 (1.011, 1.386)	723	1.163 (0.917, 1.475)	1089	1.189 (0.961, 1.470)
** Several periods**	48	0.899 (0.393, 2.056)	27	0.766 (0.251, 2.332)	21	1.115 (0.321, 3.876)
** Persistent**	114	1.387 (0.841, 2.289)	49	1.150 (0.548, 2.414)	65	1.568 (0.797, 3.086)

^a^Adjusted for age, BMI, family history of hypertension, tobacco use, intake of fatty foods, intake of vegetables and fruits, blood biochemical indicators (fasting plasma glucose, triglycerides, and high-density lipoprotein cholesterol), education, marital status, and physical activity.

^b^Also adjusted for gender.

### Sensitivity analysis for risk of hypertension by two different samples

Sensitivity analysis were conducted in two datasets using logistic regression model adjusted the same factors as in the main study (Table 4). The results of dataset 2 are very consistent with the results of the main study. However, unlike the main study analysis, the results of dataset 1 showed nonsignificant odds ratios for stress at work in the total sample (OR = 1.043, 95% CI [0.932, 1.169]) and women (OR = 1.080, 95% CI [0.923, 1.263], and nonsignificant odds ratios for stress at home in women (OR = 1.122, 95% CI [0.977, 1.289].

**Table 4:** The results of the sensitivity analysis for different datasets (odds ratios ^a^).

		Total	Men	Women
**Dataset 1** ^**b**^	**General stress**			
	** No**	1	1	1
	** Yes**	1.177 (1.064, 1.301)	1.190 (1.018, 1.390)	1.174 (1.028, 1.340)
	**Stress at work**			
	** No**	1	1	1
	** Yes**	1.043 (0.932, 1.169)	1.000 (0.848, 1.179)	1.080 (0.923, 1.263)
	**Stress at home**			
	** No**	1	1	1
	** Yes**	1.125 (1.013, 1.249)	1.133 (0.963, 1.333)	1.122 (0.977, 1.289)
**Dataset 2** ^c^	**General stress**			
	** No**	1	1	1
	** Yes**	1.216 (1.076, 1.374)	1.185 (1.083, 1.424)	1.246 (1.057, 1.467)
	**Stress at work**			
	** No**	1	1	1
	** Yes**	1.129 (1.012, 1.271)	1.051 (0.866, 1.275)	1.206 (1.018, 1.455)
	**Stress at home**			
	** No**	1	1	1
	** Yes**	1.154 (1.015, 1.309)	1.093 (0.902, 1.324)	1.205 (1.017, 1.427)

^a^Same factors as in the main study were adjusted in the logistic regression analyses.

^b^(n = 8331) whole population excluded participants who had missing stress data (from work or home) or data for one of the main demographic variables (age or gender).

^c^(n = 7160) whole population excluded participants who had missing stress data (from work or home) or data for one of the main demographic variables (age or gender), or who had taken antihypertension/lipid-lowering agents regularly.

### Population-attributable risk for hypertension by different types of stress

Table 5 shows the PAR associated with different types of stress for the different groups. Among all collected factors of hypertension in the study, general stress contributed 9.1% (95% CI [3.1, 15.0]) to the risk of hypertension in the total sample, and the PARs of stress at work and stress at home were 4.9% (95% CI [0.4, 9.4]) and 5.9% (95% CI [0.8, 10.9]), respectively. The PARs of general stress, stress at work, and stress at home were 9.4%, 2.8%, and 4.2% in men and 8.5%, 6.0%, and 6.8% in women, respectively.

**Table 5:** Population-attributable risk of hypertension by different types of stress and gender based on logistic regression^a^.

	Total^b^ (n = 5976)		Men (n = 2359)		Women (n = 3617)	
	n (%)	PAR (95% CI)	n (%)	PAR (95% CI)	n (%)	PAR (95% CI)
**General stress**	2298 (38.45)	0.091 (0.031, 0.150)	959 (40.65)	0.094 (0.001, 0.187)	1339 (37.02)	0.085 (0.007, 0.161)
**Stress at work**	1718 (28.75)	0.049 (0.004, 0.094)	810 (34.34)	0.028 (-0.051, 0.106)	908 (25.10)	0.060 (0.006, 0.113)
**Stress at home**	1974 (33.03)	0.059 (0.008, 0.109)	799 (33.87)	0.042 (-0.035, 0.118)	1175 (32.49)	0.068 (0.001, 0.135)

PAR: population-attributable risk; 95% CI: 95% confidence interval.

^a^Adjusted for age, BMI, family history of hypertension, tobacco use, intake of fatty foods, intake of vegetables and fruits, blood biochemical indicators (fasting plasma glucose, triglycerides, and high-density lipoprotein cholesterol), education, marital status, and physical activity.

^b^Also adjusted for gender.

### The interactions of gender and different types of stress

**Fig 1:**
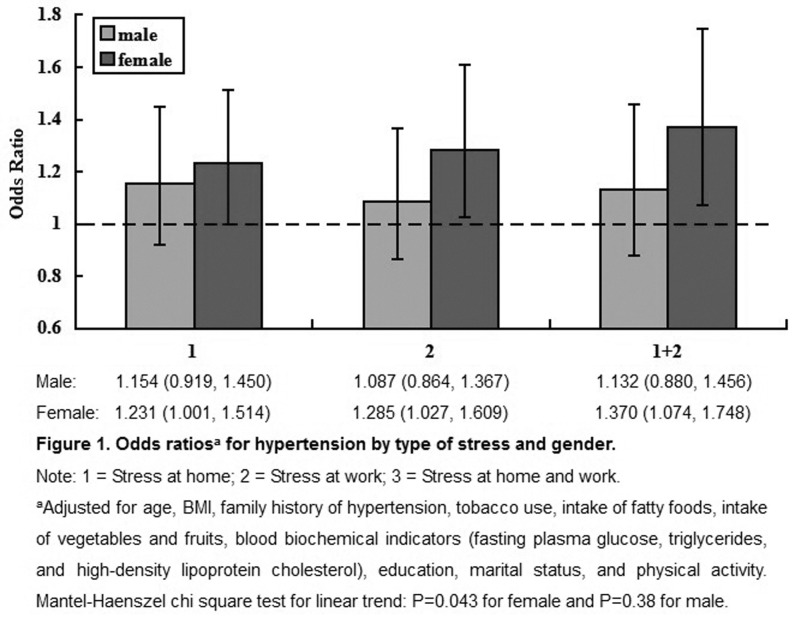
Odds ratios of hypertension by types of stress and gender. This figure shows the interaction of gender and different types of stress on the risk of hypertension. Among women with stress at work and at home simultaneously, the odds of hypertension (OR = 1.370, 95% CI [1.074, 1.748]) concomitantly increased with change of stress type (P = 0.043 for linear trend). This trend was not significant for men (P = 0.38).

The interactions of gender and different types of stress were tested by logistic regression analyses adjusted for age, BMI, family history of hypertension, tobacco use, intake of fatty foods, intake of vegetables and fruits, blood biochemical indicators (fasting plasma glucose, TG, and HDL-C), education, marital status, and physical activity. The interactions of gender with various types of stress (at work, at home, and general) were not significant. Fig 1 shows the associations of gender and different types of stress with the risk of hypertension. Both women and men who had stress at work and at home simultaneously showed a higher odds of hypertension (OR = 1.370, 95% CI [1.074, 1.748] for women, OR = 1.132, 95% CI [0.880, 1.456] for men). The results of a linear trend test showed that the rates of hypertension concomitantly increased with change of stress type (stress at work and at home simultaneously) for women (P = 0.043), but not for men (P = 0.38).

## Discussion

A number of studies have demonstrated that psychological stress is associated with increases in BP and the development of hypertension [17, 18], while other studies have failed to show a relationship [19]. Our results indicated that psychological stress at work and home was associated with an increased risk of hypertension for middle-aged Chinese participants after adjusting for the confounding variables. However, the strength and direction of the associations were not consistent across gender.

### Gender differences in physiological stress

Gender differences in response to stress have been found previously. Studies of the physiological markers of emotional responses to stress have indicated that men have a greater blood pressure response than do women, although women have a greater heart rate response [20, 21]. Ohlin et al [22] noted that the effects of job strain on BP tend to be stronger among men than among women. However, we found that Chinese middle-aged women appeared to be more susceptible to psychological stress than did men. We proposed two reasons for this difference in findings. First, men and women were engaged in different types of work, and the specific type of work may be related to specific reactions. For example, in a longitudinal study, low-status work predicted hypertension among women, whereas job insecurity and low self-reported job performance each independently predicted the incidence of hypertension in men [23]. Second, the different effects of psychological stress may be related to sociocultural background. In China, people are still influenced by traditional ideology, in which men and women are not equal in either social or economic status. Although economic development has greatly changed this situation, many women remain in subordinate positions and are often not independent, especially in rural areas. In such environments, women may be more likely to experience intensive influences and have a higher risk of functional disorders related to psychological stress than men may. Clearly, cultural differences should be considered when comparing the impact of stress across different populations. It is worth noting that there was an interaction between stress at work and stress at home with an increased hypertension risk for women. This phenomenon was not found in men and is another indication that women in China may be more vulnerable than men may be to the effects of psychological stress.

### Potential mechanisms

Previous animal and human studies have comprehensively explained what psychological stress mechanisms induce the increase in BP. However, reactions to psychological stress are diverse and complex and differ between men and women. In general, women have a higher prevalence of affective disorders, whereas men are more prone to substance use disorders [24]. The main line of research on gender differences relates to the organizational and activational role of gonadal hormones, which appear to affect neurotransmitter systems. The higher levels of hypothalamic-pituitary-adrenal (HPA) axis hormones found in female rodents and in some human studies suggest greater activation of the HPA axis, which may be due to the presence of corticotrophin releasing factor (CRF) in women. Some studies have reported that female rodents have increased CRF expression in the paraventricular nucleus (PVN) of the hypothalamus under basal conditions, particularly when estrogen levels are high [25–27]. In contrast, restraint stress and chronic mild stress increase CRF messenger RNA (mRNA) in the PVN to a larger degree in males than in females, and this may be because females have a higher basal CRF expression, which could obscure the increase in CRF mRNA [25, 27–29]. On the other hand, some studies have reported that dopamine neurons in the ventral tegmental area (VTA) react strongly to stressful situations [30, 31]. Trainor [32] found that neurobiological and behavioral responses to stress are frequently gender-dependent. Finally, the renin-angiotensin system is intimately involved with the stress response, and angiotensin II is a major stress hormone, similar to glucocorticoids and catecholamines [33]. Aguilera et al. [34] demonstrated that chronic intermittent stress significantly increases plasma renin activity (PRA) and plasma aldosterone concentration, and acute stress had the same effect. However, evidence of gender differences in the renin-angiotensin system response to stress has not been consistent.

### Contribution of psychological stress to health

In this study, we found that psychological stress contributed about 9% to the risk of hypertension. A case-control study from 52 countries [35] reported strong associations of myocardial infarction with frequent periods of home-based stress, severe financial stress, and a greater number of stressful life events. Among the risk factors for myocardial infarction, the effect of psychosocial stress was as important as those of traditional CVD risk factors, such as smoking, obesity, and diabetes. To the best of our knowledge, the risk magnitude of psychological stress for chronic diseases has been evaluated in only a few previous studies, and the influence of psychological stress on general health may become more conspicuous with the lifestyle changes caused by the technological revolution. Therefore, the contribution of psychological stress to general health must be further investigated using different methods in future studies.

### Strengths and limitations

The strengths of our study are our use of quality control methods throughout the study protocol. Namely, trained research assistants used uniform standardized methods of data collection and laboratory testing, and data on most of the traditional risk factors of hypertension were comprehensively collected.

In the current study, we included only participants without a history of hypertension or CVD and who were not regularly taking antihypertension/lipid-lowering agents, as this would reduce the likelihood of our results being affected by previous hypertension and CVDs substantially altering participants’ lifestyles and risk factor levels. Nevertheless, we performed two more analyses to test the sensitivity of the results using different datasets. The only difference between the two datasets is the exclusion of those who were regularly using antihypertension/lipid-lowering agents. It has been known that use of such agents is one of the most serious confounders in studies of chronic diseases (e.g., CVD, diabetes). Using logistic regression analysis and adjusting for the same factors, we found in both datasets similar results: that psychological stress was associated with an increased risk of hypertension. The results of the sensitivity analyses show that the findings of our study are robust and credible.

Few studies have explored the association between psychological stress intensity and hypertension. In our study, for both genders, “persistent” stress at work and at home, which was the most severe stress type in our study, was not found to be significantly associated with risk of hypertension. Of the total sample of nearly 6,000 in analyses, we found that only 100 and 114 participants have “persistent” stress at work and at home, respectively. Moreover, it has been observed that the CI values of hypertension risk for “persistent” stress at work and at home were extended to a wider margin. Therefore, it is possible that an insufficient sample size was the main reason for these nonsignificant results. However, hypertension risk did appear to increase as stress was aggravated, as shown by ORs and CI values for different degree of stress, even if these results were not significant. The association of psychological stress intensity and hypertension must be investigated in larger samples.

There were several other limitations as well. First, the study was cross-sectional in design; therefore, causal relationships cannot be inferred. Second, some selection bias might have occurred because participants were only recruited among community members aged 40–60 years. The influence of psychological stress on blood pressure may be different across ages. Third, only fatty foods (meats, fish, eggs, and milk) and vegetables and fruits were included in the examination of diet, but other types of foods (e.g., nuts, edible oils, sweet drinks, snacks) were not. The other shortcoming in this area was that a verified intake of salt by the participants was not collected. These limitations could affect the accuracy of the study results and the scope of our conclusions.

## Conclusions

Psychological stress (including stress at work and at home) was associated with a greater risk for hypertension in a middle-aged Chinese sample, and stress could account for about 9% of the risk of hypertension. In addition, psychological stress contributed to a greater risk for hypertension in women than in men. This gender difference in response to psychological stress requires further exploration.
